# Long-term prediction modeling of shallow rockburst with small dataset based on machine learning

**DOI:** 10.1038/s41598-024-64107-3

**Published:** 2024-07-12

**Authors:** Guozhu Rao, Yunzhang Rao, Jiazheng Wan, Qiang Huang, Yangjun Xie, Qiande Lai, Zhihua Yang, Run Xiang, Laiye Zhang

**Affiliations:** 1 School of Emergency Management and Safety Engineering, Jiangxi University of Science and Technology, Ganzhou, 341000 China; 2 Ganzhou Key Laboratory of Industrial Safety and Emergency Technology, Jiangxi University of Science and Technology, Ganzhou, China; 3 School of Resources and Environmental Engineering, Jiangxi University of Science and Technology, Ganzhou, 341000 China; 4 Jiangxi Provincial Key Laboratory of Low-Carbon Processing and Utilization of Strategic Metal Mineral Resources, Jiangxi University of Science and Technology, Ganzhou, China

**Keywords:** Natural hazards, Engineering, Mathematics and computing

## Abstract

Rockburst present substantial hazards in both deep underground construction and shallow depths, underscoring the critical need for accurate prediction methods. This study addressed this need by collecting and analyzing 69 real datasets of rockburst occurring within a 500 m burial depth, which posed challenges due to the dataset's multi-categorized, unbalanced, and small nature. Through a rigorous comparison and screening process involving 11 machine learning algorithms and optimization with KMeansSMOKE oversampling, the Random Forest algorithm emerged as the most optimal choice. Efficient adjustment of hyper parameter was achieved using the Optuna framework. The resulting KMSORF model, which integrates KMeansSMOKE, Optuna, and Random Forest, demonstrated superior performance compared to mainstream models such as Gradient Boosting (GB), Extreme Gradient Boosting (XBG), and Extra Trees (ET). Application of the model in a tungsten mine and tunnel project showcased its ability to accurately forecast rockburst levels, thereby providing valuable insights for risk management in underground construction. Overall, this study contributes to the advancement of safety measures in underground construction by offering an effective predictive model for rockburst occurrences.

## Introduction

Rock explosion is a significant hazard within underground engineering operations^1,2^, characterized by the sudden release of energy within the rock mass due to high stress conditions induced by excavation, mining activities, or external disturbances^3^. This phenomenon can result in various complex geological disasters such as bursting, stripping, ejection, or even expulsion of rock fragments. While there exists a general understanding that the likelihood of rock explosion accidents increases with the depth of burial, this correlation is not absolute. Extensive research and analysis of rock explosion engineering cases have revealed that shallow engineering operations are also susceptible to such occurrences^4,5^.

The occurrence of rock explosions poses a grave threat to the safety of operational workers and presents a significant challenge to the pursuit of efficient and safe production practices, particularly within the context of underground engineering projects in China^6–8^. Consequently, there is an urgent need for comprehensive research and analysis aimed at predicting and preventing rock explosion disasters. Addressing this imperative is paramount for ensuring the continued and healthy development of underground engineering endeavors, given the critical importance of safety considerations in such operations^9–11^.

In recent years, numerous researchers have developed a plethora of models aimed at assessing the intensity levels of rockburst^12,13^. Sun et al.^14^ proposed a short-term rockburst prediction model based on microseismic monitoring and probabilistic optimized plain Bayes. Qiu et al.^15^ proposed a new hybrid model based on extreme gradient boosting (XGB) and meta-heuristic sand cat swarm optimization (SCSO). Liang et al.^16^ proposed an integrated classifier based on five basic learners to obtain better prediction results. Sun et al.^17^ showed that the prediction of rockburst and rockburst based on 13 machine learning algorithms is more accurate than a single model. Li et al.^18^ proposed a new method combining t-distributed stochastic neighborhood embedding (t-SNE) and Gaussian Mixture Model (GMM) clustering to relabel the dataset which can effectively improve the model prediction accuracy and generalization ability. Barkat et al.^19^ proposed an algorithmic combination of t-SNE, K-Means clustering, and XGBoost to predict the rockburst intensity level, which provides a good benchmark for future high-accuracy modeling. Muhammad et al.^20^ developed an ISOMAP + FCM + KNN framework that allows for high-precision prediction of rockbursts for short periods of time under specific conditions.

While recent advancements in model performance have undoubtedly contributed to theoretical development, there are lingering areas for improvement. Specifically, many existing models overlook the influence of burial depth on accuracy and expend considerable resources on hyper parameter optimization. Variations in burial depth can induce changes in ground stress, thereby altering rock explosion dynamics. Consequently, partitioning the dataset by burial depth becomes imperative to enhance model accuracy and applicability. Optuna^21^ emerges as a promising avenue in automated machine learning, offering efficiency gains over traditional grid and random search methods by swiftly determining optimal hyper parameter combinations. Overall, this paper presents a low-cost and efficient model by collecting data through the changing trend of the stress state at a certain depth of burial and innovatively applying Optuna, which highlights the high accuracy and practicality compared with other mainstream rockburst models. In this study, it meticulously curated 69 sets of real rock burst case parameters occurring within a 500 m burial depth range from literature sources. Employing KMeansSMOTE oversampling technology^22^, this study address data imbalances, subsequently optimizing the Random Forest^23^ integration model with Optuna to establish the KMSORF integration model. The efficacy and accuracy of our approach are validated through empirical investigations conducted in a tungsten mine and a tunnel project.

The structure of this paper is delineated as follows: The Background section provides a comprehensive introduction to the study's context and objectives. In the Rock Burst Data Collection and Analysis section, this study meticulously gather and analyze relevant datasets, illustrating correlations and delineating general characteristics through graphical representations. The subsequent section on KMeansSMOTE oversampling processing and analysis delves into algorithmic intricacies, particularly focusing on its efficacy in enhancing model performance with small datasets. This study then detail the optimization process of the KMSORF integration model using Optuna in the subsequent section. This is followed by a comprehensive explanation of the Optuna-based Random Forest Algorithm, elucidating its principles and the iterative steps involved in model training. In the Evaluation of the Model and its Application section, this study rigorously assess our model's performance against existing benchmarks, affirming its superior accuracy and reliability through comparative analyses and real-world case studies. In the Strengths and Limitations section, make a critical evaluation, citing the corresponding innovations while pointing out the limitations. Finally, in the Conclusion section, this study synthesize key insights and propose avenues for future research, encapsulating the contributions and significance of our work.

## Shallow rockburst data acquisition and analysis

This study presented an analysis of 69 instances of shallow rock burst engineering examples^24–30^, each devoid of missing values and occurring within a burial depth of 500 m. Drawing upon a comprehensive review of literature from both domestic and international sources^17,18,31–36^, machine learning techniques and comprehensive evaluations of rock burst factors were employed. Specifically, the investigation focused on stress control, rock mechanical properties, and energy considerations. Through a number of case studies it can be seen that the intensity of rock bursts occur at the level of the following characteristics: occurred in the stress concentration area, the form of section damage was mainly tensile damage accompanied by shear damage, can store a large amount of elastic energy and other salient features. At the same time, the indicators chosen should be common, easy to measure in practice, and documented in previous examples of rockburst. So Four key indicators were selected for rock burst prediction characterization, namely the maximum tangential stress of the surrounding rock (${\sigma }_{\theta }$), uniaxial compressive strength (${\sigma }_{c}$), uniaxial tensile strength (${\sigma }_{t}$), and elastic energy index (${W}_{et}$). These indicators served as crucial features for predicting rock burst intensity levels, categorized as None, Light, Moderate, or Strong based on actual occurrences.

**Figure 1:**
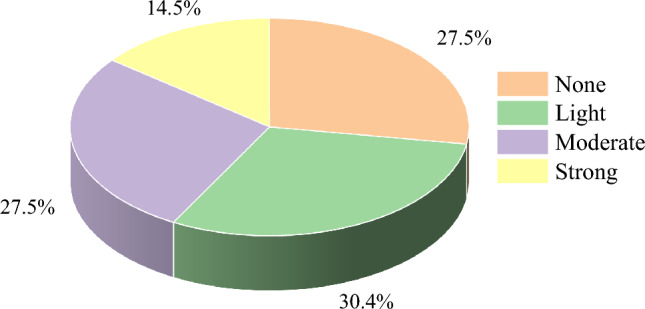
Sample dataset share chart.

In the dataset comprising 69 rockburst samples, the distribution across rockburst grades reveals 19, 21, 19, and 10 instances categorized as none, weak, moderate, and strong, respectively. Notably, the proportion of strong-level rock burst data was relatively small, as illustrated in Fig. 1. To address this imbalance, this study proposed oversampling the dataset to ensure a more equitable representation across all intensity levels.

**Figure 2:**
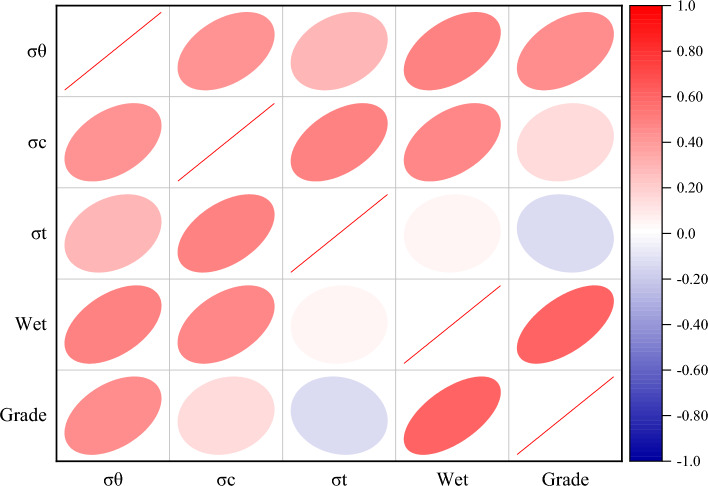
Pearson's correlation coefficient plot.

To elucidate the horizontal relationships between the indicators, this study calculated the Pearson correlation coefficient heat map, presented in Fig. 2. The analysis underscores a strong correlation between the indicators and the intensity of rockburst grades. Specifically, heightened values of rock elasticity, energy index, and surrounding rock stress correspond to an elevated likelihood and intensity of rock burst occurrences.

**Figure 3:**
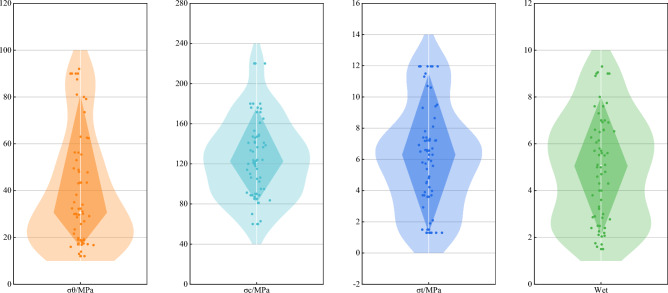
Violin case line diagram.

Furthermore, to illustrate the longitudinal relationships of the characteristic indicators more vividly, this study depicted the data distribution using violin box line graphs, as shown in Fig. 3. The analysis reveals that ${\sigma }_{\theta }$, ${\sigma }_{c} {,\sigma }_{t}$ and ${W}_{et}$ predominantly fall within the ranges of 20–80 MPa, 80–180 MPa, 2–10 MPa and 2–8, shedding light on the distribution patterns of these crucial factors.

## KMeansSMOTE oversampling processing and analysis

The KMeansSMOTE oversampling method^22^, introduced by Georgios D, Fernando B et al. in 2018, represented a novel algorithm designed to address sample imbalance. Initially, the algorithm employs KMeans clustering to partition the original unevenly distributed samples into k clusters. These filtered clusters are denoted as f. Subsequently, the sample size for each group of generated data was computed based on the weights of sample categories. Finally, the filtered clusters undergo oversampling using the Synthetic Minority Over-sampling Technique (SMOTE).This methodology offered a systematic approach to mitigate sample imbalance, thereby enhancing the robustness and reliability of classification models. The specific calculation process unfolds as follows:

1. Input the original unbalanced k clusters by clustering, calculate the degree of unbalance for each cluster, and if it is greater than a specified threshold then select this cluster noted as *f*. *k* can be based on:
  1 $$k = \root 2 \of {\frac{N}{2}}$$
  where *N* is the total number of samples.
2. Calculate the density of cluster *f*:
  2 $$D(f)=\frac{MC(f)}{\overline{d}}$$
  where $MC(f)$ is the number of minority samples in the cluster and $\overline{d}$ is the average distance within the cluster.
3. Compute the sparsity of the cluster *f*:
  3 $$S(f)=\frac{1}{D(f)}$$
4. Calculate the sampling coefficients of the clusters *f*:
  4 $$W({f}_{i})=\frac{S(f)}{\sum_{i=1}^{n}S(f)}$$
  where n is the number of clusters selected for synthesizing new samples.
5. Calculate the number of new samples to be synthesized artificially based on the sampling coefficients and the m samples generated:
  5 $$G({f}_{i})=m\times W({f}_{i})$$
6. Finally, the Smote algorithm is used to generate new minority class samples based on within clusters.
  6 $${X}_{new}=G({f}_{i})+rand(\text{0,1})\left|x-{x}_{i}\right|$$
  where ${X}_{new}$ is a newly synthesized minority class sample and is a random number within (0, 1).

Numerous studies have underscored the profound impact of sample category imbalance on model performance^37^. Generally, three approaches are commonly employed to address the data imbalance issue: undersampling, oversampling, or a combination of both. Given the constraints imposed by limited sample numbers, this study opts for the oversampling method.

KMeansSMOTE represents an innovative oversampling technique that integrates the K-means clustering algorithm with the SMOTE. This approach effectively addresses challenges associated with inadequate sample data across categories and enhances model generalization capabilities. Consequently, it contributes to improving the accuracy and robustness of the model. This methodological choice aligns with the objective of mitigating data imbalance to facilitate more reliable and insightful model outcomes.

To assess the sensitivity and generalization capabilities of various algorithms on unbalanced small datasets, as well as to evaluate the efficacy of the KMeansSMOTE oversampling method in enhancing model performance, this study conducted a comparative analysis of prediction results using eleven machine learning models. The comparison involved evaluating the original dataset against both normalized and pre-processed datasets after KMeansSMOTE oversampling. To ensure consistency, the model hyper parameter was optimized using GridSearchCV, thereby ensuring identical hyper parameter values across datasets. Cohen's Kappa coefficients were employed as a metric for evaluation.

The eleven machine learning models considered in this study were: Decision Tree (DT), Support Vector Classification (SVC), k-Nearest Neighbor (KNN), Gaussian Process Regression (GPR), Naive Bayes model (NBM), Quadratic Discriminant Analysis Algorithm (QDA), Gradient Boosting (GB), Extreme Gradient Boosting (XGB), Random Forest (RF), Extra Trees (ET), Light Gradient Boosting (LGB), and Light Gradient Boosting Machine (LGBM).

This rigorous comparative analysis aims to provide insights into the performance variations among different algorithms and the impact of oversampling techniques on model efficacy. By systematically evaluating these models across various datasets and conditions, this study aim to validate the effectiveness of the proposed approach and contribute to advancing the understanding of handling unbalanced small datasets in machine learning applications.

**Figure 4:**
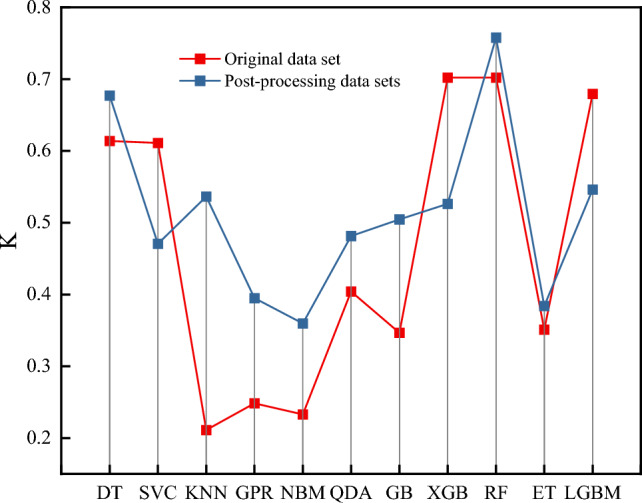
Performance of KMeansSMOTE algorithm in different models.

As depicted in Fig. 4, the utilization of KMeansSMOTE oversampling demonstrates a significant enhancement in model performance. Across multiple models, the coefficients K on the original dataset average 0.4639, while those on the post-processed dataset average 0.5127. Notably, 8 out of 11 algorithms exhibit improved performance, with KNN demonstrating the highest improvement at 0.3252. In the post-processed dataset, the RF model achieves the highest score of 0.7578.

Moreover, the integrated model displays a notable advantage over individual models, exhibiting an improvement of approximately 0.1693 in the original dataset and 0.0571 in the processed dataset. This observation underscores the superior performance of the integrated model compared to individual models.

Overall, the results highlight the effectiveness of the KMeansSMOTE algorithm in enhancing individual model performance. However, it is noteworthy that the integrated model outperforms both the single model and the KMeansSMOTE algorithm alone, underscoring the importance of model integration for achieving superior predictive accuracy. These findings contribute to a deeper understanding of model enhancement techniques and their impact on overall performance in machine learning applications.

## Random forest algorithm based on Optuna

**Figure 5:**
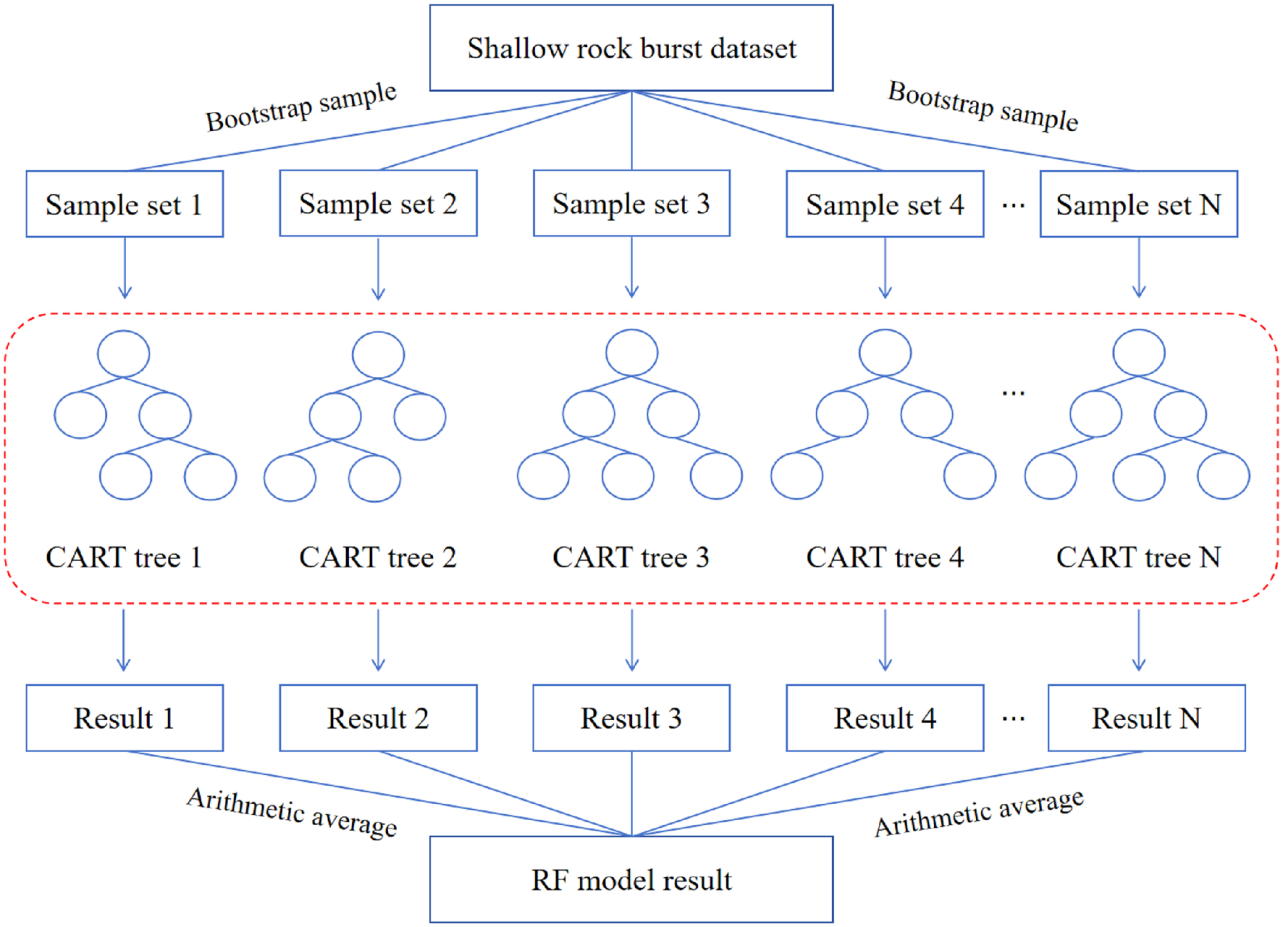
Principle diagram of RF.

Random Forest^38^ (RF) was a machine learning algorithm rooted in the concept of Bagging integrated learning, aimed at constructing multiple weak classifiers and amalgamating them to form a robust classifier. Within RF, the weak classifiers typically comprised Classification and Regression Trees (CART). During the construction of CART trees, training samples were subjected to a self-servicing sampling method known as Bootstrap Sample, ensuring that each CART tree possesses independent features and judgment criteria. Moreover, a subset of features was randomly selected for training, fostering diverse combinations of features and enhancing generalization capability. This approach not only enhances diversity but also improves the algorithm's generalization ability, thereby facilitating more accurate and robust predictions. These principles underpin RF efficacy in various machine learning tasks and contribute to its widespread adoption in scientific research and practical applications.The detailed process is visually delineated in Fig. 5. The RF integrated classification model can be expressed as follows:

7 $$H(x)=arg\underset{Y}{\mathit{max}}{\sum }_{i=1}^{k}I({h}_{i}(x)=Y)$$

where $H(x)$ is the integrated classification model, *Y* is the output variable, ${h}_{i}$ is the single CART tree classification result, and $I$ is the indicator function.

This study uses the Tree-structured Parzen Estimator sampler (TPESampler) improved by the Bayesian optimization algorithm in Optuna, whose substitution probability model is a Gaussian process distribution model determined by the mathematical expectation and covariance functions, which can be expressed as follows:

8 $$f(x)\sim P(m(x),k(x,x{\prime}))$$

where any x corresponds to the probability density function as a normal distribution function, m is the mathematical expectation, and k is the covariance function.

The value of the kernel function $k(x,x{\prime})$ is crucial, as it determines the similarity between function values and controls the shape of the fitted function. Assuming $m(x)$=*0*, a number of ${X}_{1:t}$ are taken uniformly and brought into $f(x)$ to obtain:

9 $$\left[\begin{array}{c}\varphi ({X}_{1:t})\\ \varphi ({X}_{1+t})\end{array}\right]\sim N \left (\left[\begin{array}{c}0\\ \vdots \\ 0\end{array}\right],\left[\begin{array}{cc}K& k\\ {k}^{T}& k({x}_{1+t},{x}_{1+t})\end{array}\right] \right)$$

where:

10 $$k=[k({x}_{t+1},{x}_{1}),k({x}_{t+1},{x}_{2})\ldots k({x}_{t+1},{x}_{t})]$$

where $\varphi ({X}_{1:t})$ is the output obeys a multivariate Gaussian distribution with mean 0 and covariance matrix K, and ${X}_{1+t}$ is the new sampling point for the search.

This leads to suit $\varphi ({X}_{1+t})$ to compute the new sampling point function values from a one-dimensional normal distribution. A Gaussian process is fitted to the prior function to find the probability distribution of $\varphi ({X}_{1+t})$ function values:

11 $$P(\varphi ({X}_{1+t}|{X}_{1:t},{x}_{1+t}))=\frac{1}{\sqrt{2\pi }{\sigma }_{1+t}}{e}^{-\frac{{(x-{u}_{1}+t)}^{2}}{2{\sigma }_{1+t}^{2}}}$$

The Gaussian process described above can be modeled as $p(x|y)$. The tree-structured Parzen estimator replaces the previously configured distribution transformation generation process with a nonparametric density to model $\text{p}(\text{x}|\text{y})$, which can be expressed as:

12 $$p(x|y)=\left\{\begin{array}{ll}\delta (x), & \quad y<{y}^{*}\\ g(x), & \quad y\ge {y}^{*}\end{array}\right.$$

where ${y}^{*}$ is the best value searched, $\delta (x)$ is the density formed by different $x$, and $g(x)$ is the density formed by the remaining search values.

The RF algorithm entails adjusting numerous hyper parameter, and manual tuning can be time-consuming and prone to issues such as local optima. Moreover, manual tuning does not guarantee optimal prediction results. Thus, this study employed Optuna to swiftly and effectively determine optimal values. The methodology unfolds as follows:

Step 1: Data preprocessing involves normalization, segmentation, and preprocessing of collected data. Following processing, the dataset is partitioned into training and test sets in an 8:2 ratio.
Step 2: Identification of hyper parameter to be tuned in the RF algorithm. Given the multitude of hyper parameter, focus is placed on those with significant impact, including the number of CART trees (n_estimators), maximum depth of CART tree (max_depth), minimum samples required to split internal nodes (min_samples_split), and whether to utilize bootstrapping (bootstrap). Other hyperparameters retain their default values initially. The ranges of hyper parameter values are determined based on literature review and preliminary testing, as outlined in Table 1.

**Table 1:** RF algorithm hyper parameter optimization range and determining hyper parameter values.

Hyper parameter	Limit	Lower limit	Optuna auto-adjusted values
n_estimators	2500	500	1973
max_depth	50	10	None
min_samples_split	10	1	2
min_samples_leaf	5	1	2
bootstrap	–	–	False
Step 3: Configuration of key modules in the TPESampler optimizer. This involves defining the search space for hyper parameter, setting the optimization metric (e.g., accuracy), specifying the objective function, and determining the number of trials. For this study, the default number of trials is set to 50.
Step 4: Determination of optimal hyper parameter values. Optimization is based on accuracy and employs four-fold cross-validation for evaluation. The algorithm's performance is compared with other mainstream integration algorithms on both training and test sets.
Step 5: Construction of a long-term rockburst prediction model for KMSORF. The KMSORF model classifier is trained on the training set using the hyper parameter combination identified in step 4.

This methodology ensures efficient hyper parameter optimization and robust model development, thereby advancing the field of rockburst prediction modeling.

## Assessment models and applications

### Model evaluation

After establishing the rockburst prediction model, it's crucial to select appropriate metrics for performance evaluation. Multi-classification models are typically assessed using Cohen's Kappa coefficient (K)^39^, Accuracy (acc), and Macro-averaged metrics such as Macro-Precision, Macro-Recall, and Macro-F1 Score. However, relying solely on arithmetic averages to derive these statistical indicators for each classification can potentially overlook sample imbalances. To address this issue, this study adopt the Weighted method, encompassing Weighted-Precision (WP), Weighted-Recall (WR), and Weighted-F1 Score (WF). Here, L represents the number of specific samples in the multi-classification sample, and each evaluation index is computed using the following formulas. This approach ensures a more nuanced consideration of sample imbalances, enhancing the robustness of this model evaluation process.

13 $$K=({p}_{e}-{p}_{h})/(1-{p}_{h})$$

where ${p}_{e}$ is the percentage of correctly categorized samples and ${p}_{h}$ is the consistency of labels when randomly assigning categories.

14 $$acc=\frac{T{P}_{i}+{TN}_{i}}{T{P}_{i}+{TN}_{i}+{FP}_{i}+{FN}_{i}}$$

15 $$WP=\frac{{\sum }_{i=1}^{L}\frac{T{P}_{i}}{T{P}_{i}+{FP}_{i}}\times {w}_{i}}{\left|L\right|}$$

16 $$WR=\frac{{\sum }_{i=1}^{L}\frac{T{P}_{i}}{T{P}_{i}+{FN}_{i}}\times {w}_{i}}{\left|L\right|}$$

17 $$WF=\frac{2 \cdot Weighted \; Precision\cdot Weighted \; Recall}{Weighted \; Precision+Weighted \; Recall}$$

where the values are taken according to Table 2.

**Table 2:** Confusion matrix.

Predict	Actual	
	Positive	Negative
Positive	TP_i_	FP_i_
Negative	FN_i_	TN_i_

The KMSORF model proposed in this study is compared with four mainstream "tree" model integration algorithms, namely GB, XBG, and ET. Hyper parameter optimization for these comparison algorithms is conducted using RandomizedSearchCV, ensuring robustness in model parameter selection. Four-fold cross-validation is employed to determine the specific parameter values, thereby enhancing the generalization performance of the models. The specific parameters for each algorithm are as follows: In GB, subsample = 0.8, n_estimators = 2289, min_samples_split = 2, min_samples_leaf = 2, learning_rate = 0.6; In XBG, max_depth = 10, learning_rate = 0.094, subsample = 0.911, colsample_bytree = 0.467, min_child_weight = 1; In ET, n_estimators = 138, min_samples_split = 13, min_samples_leaf = 7, max_features = sqrt, max_depth = 10. Other hyper parameter values are kept at their default settings. This rigorous parameter optimization process ensures the comparability and reliability of the model evaluations conducted in this study.

**Figure 6:**
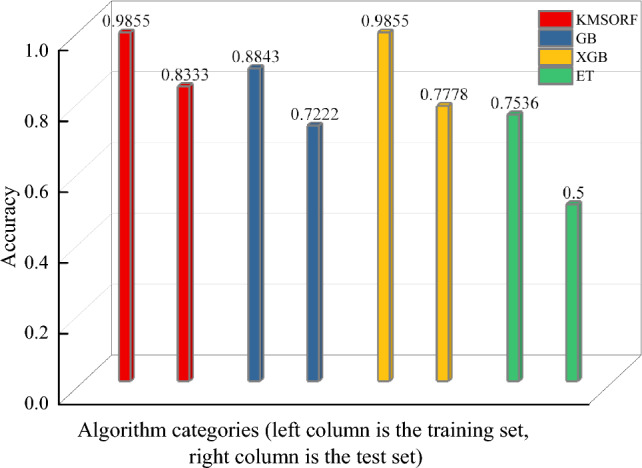
3D side-by-side bar graphs for four models accuracy.

The overall accuracy of each model is illustrated in Fig. 6. Upon examining all four models collectively, it becomes evident that both the KMSORF and XGB models exhibit superior accuracy on the training set compared to the other two integrated models, achieving an impressive 0.9855. This signifies the effectiveness of these two models in capturing the underlying features within the training data. On the test set, the KMSORF model achieves the highest accuracy of 0.8333, further affirming its efficacy. However, relying solely on accuracy metrics may not suffice for a comprehensive model assessment. Therefore, it is imperative to employ more comprehensive evaluation metrics to further scrutinize the models' performance.

**Figure 7:**
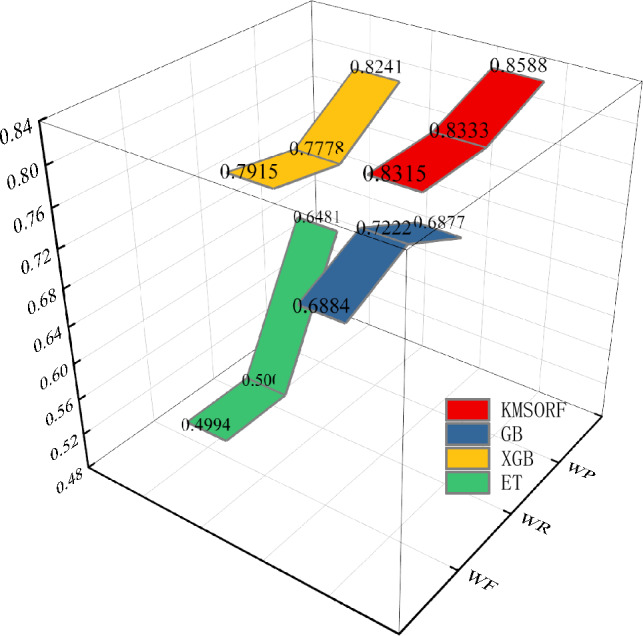
3D band chart of three indicators.

Figure 7 delineates the specific performance of the four models concerning F1 score, recall, and precision on the test set. Notably, a substantial variance among the models is observed across these evaluation metrics. Remarkably, the KMSORF model demonstrates superior performance across all three evaluation indexes, with an F1-score of 0.8315, Recall of 0.8333, and Precision of 0.8588, all of which outperform the mainstream models. These metrics collectively underscore the model's commendable predictive performance and generalization capability. The closest competitor, the XGB model, exhibits excellent performance in various domains; however, it displays certain deficiencies in generalization for smaller datasets. This nuanced analysis highlights the KMSORF model's efficacy and its potential to excel even in challenging scenarios with limited data.

### Engineering overview and applications

Overall, the value of the model needs to be demonstrated in actual engineering. In this section, the model is applied through and specific real cases in order to highlight the effectiveness and value of the model. In this study, the model is simulated and predicted by a tungsten mine in southern Jiangxi Province and a tunneling project in the cited heavy hills and mountains to determine whether the model is effective or not.

Located in the southern region of Jiangxi Province, a tungsten mine boasts an ore body primarily situated underground between depths of + 260 m and + 660 m. The terrain surrounding the mine is characterized by steep, mountainous landscapes, forming a distinctive "V"-shaped topography. The ground surface exhibits slopes exceeding 45°, extending predominantly from the northwest to the southeast. The surrounding rock formations within the mine tunnel comprise semi-hard phyllite, slate, or hard metamorphic sandstone, with occasional exposures of hidden granite in deeper sections.

Geological investigations reveal the presence of a dorsal fold structure with an axial orientation of 40° within the mining area. The most prominent fracture in the vicinity, denoted as F1, exhibits a striking trend of 70°, trending southeast, with a steep dip angle of 83°. This fracture has contributed to the formation of a crushed zone with a maximum width of 27 m.

**Figure 8:**
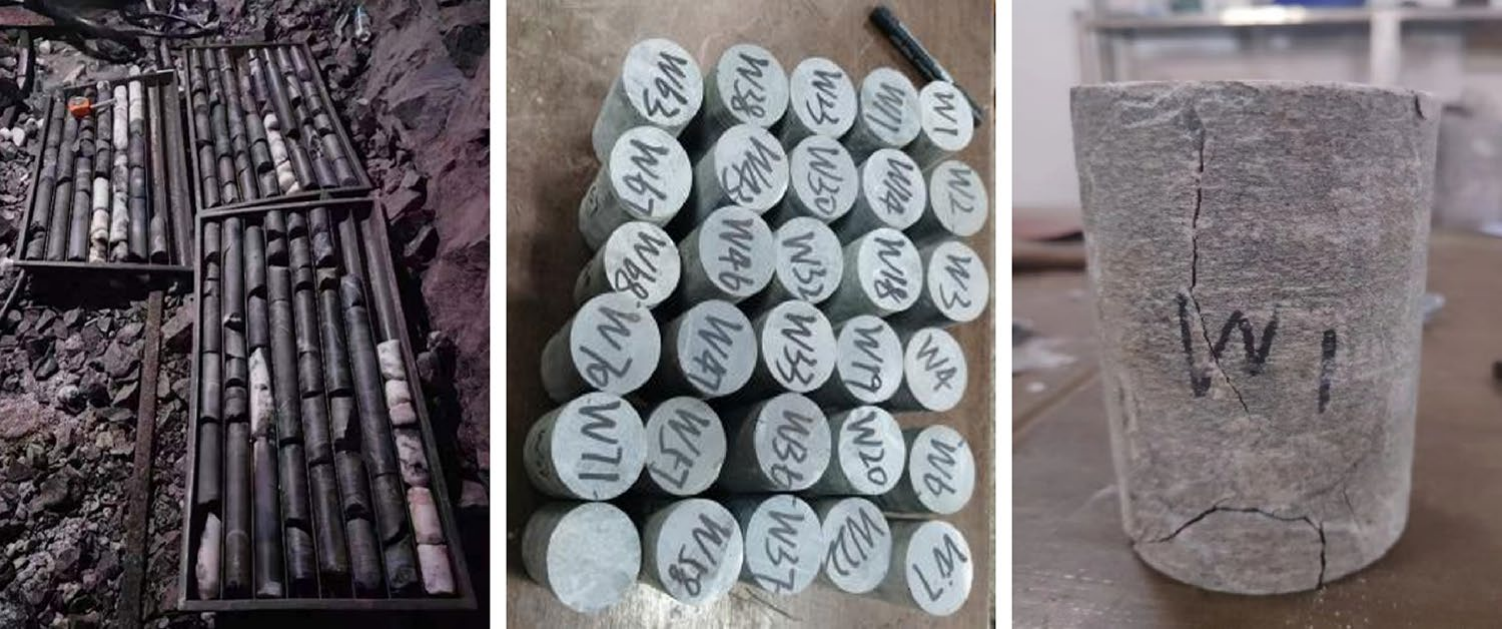
Mechanical parameter test part diagram.

To comprehensively analyze the geological conditions surrounding rock blasting operations in the mine periphery, extensive experiments have been conducted to investigate the main lithology, strength, and mechanical parameters of the granite formations within the mine. Figure 8 illustrates a subset of the mechanical parameters examined, while the comprehensive test findings are meticulously presented in Table 3. These results play a pivotal role in advancing this study understanding of the lithological granite's mechanical characteristics within the mine site.

**Table 3:** Physical and mechanical parameters of mine surrounding rock.

Rock group	Densities	Compressive strength	Modulus of elasticity	Poisson's ratio	Softening factor	Tensile strength	Cohesion	Friction angle
	g/cm^3^	MPa	GPa		%	MPa	MPa	°
Perimeter rock	2.695	127.780	25.391	0.250	80.200	11.757	24.075	29.061

Based on the actual geological conditions of the tungsten mine and leveraging the KMSORF rockburst prediction model, typical geological scenarios within the mine were selected for rockburst prediction. Specifically, the rock layer predominantly consists of granite, with a burial depth of 380 m. Additionally, the model was applied to predict rockburst occurrences in a tunnel project situated in Heavy Hills and Mountains^40^. In this project, the rock layer primarily comprises gray rock, with a burial depth of 410 m. The mechanical parameters and prediction results are summarized in the following Table 4.

**Table 4:** Engineering measurement data and validation results.

Project name	Lithology	*h/m*	${\sigma }_{\theta }$/MPa	${\sigma }_{c}$/MPa	${\sigma }_{t}$/MPa	${W}_{et}$	Actual grade	Forecast level
A tunnel in the Heavy Hills and Mountains	limestone	410	12.7	190	8.9	7.1	None	None
A tungsten mine in southern Jiangxi	Granite	380	37.9	127.8	11.8	6.8	None	None

The prediction outcomes indicate a congruence between the model's predictions and the actual rockburst grades, affirming the accuracy of the model. This validation of the prediction results provides robust evidence of the model's reliability and its applicability to diverse geological settings.

## Strengths and limitations

Compared to existing studies, this study introduces several notable innovations. Firstly, this study employ the novel technique of Optuna automatic hyper parameter adjustment, which effectively reduces model training time, enhancing efficiency without compromising performance. Secondly, this study distinguishes between shallow rockburst and other forms of rockburst, providing a fresh perspective on the prediction of shallow rockburst occurrences. This differentiation enriches predictive models and improves their accuracy in assessing specific geological conditions. Thirdly, this evaluation reveals the KMSORF model as the top performer among mainstream integrated models, particularly in small dataset scenarios, showcasing its superiority in predictive accuracy and generalization. The accuracy of the model in this study is 0.8333, compared to 0.8 for mainstream models used for rockbursts with small datasets (e.g., FA-SSA-PNN Model^41^). It highlights the superiority of the present model.

However, despite these advancements, certain limitations persist. Chief among them is the challenge posed by small sample sizes, necessitating a greater influx of real rockburst data to augment model training and validation. Addressing this limitation will be crucial for further refining and validating predictive models in real-world applications.

## Conclusion

In this study, this study investigated a long-term prediction model for shallow rockburst using machine learning techniques, utilizing data gathered from literature sources. Through a series of algorithmic combinations and enhancements, this study found that the KMSORF model performs exceptionally well. The key conclusions drawn from this study are as follows:

The optimized KMSORF model, incorporating the KMeansSMOTE oversampling algorithm and the Optuna learning framework on the RF base algorithm, demonstrates remarkable efficacy in long-term prediction of shallow rockburst. The model achieves superior performance metrics compared to other mainstream models, with acc reaching 0.8333, and WP, WR, and WF values of 0.8588, 0.8333, and 0.8315, respectively. Application of the model to tungsten mining in southern Jiangxi Province and tunneling projects in hilly terrains yields predictions consistent with real-world outcomes, thereby validating the reliability and accuracy of the model. These findings suggest that the model can serve as a valuable tool for guiding exploration, design, and safe construction practices in shallow underground engineering projects.

The KMeansSMOTE algorithm effectively transforms data structures, leading to performance improvements across most models. While the enhancement for individual models surpasses that of integrated models, the latter exhibit superior overall performance both before and after dataset processing. Notably, among the 11 machine learning algorithms assessed, the RF algorithm achieves the highest K-factor of 0.7578. Additionally, the KNN model demonstrates the most significant improvement post-oversampling treatment by the KMeansSMOTE algorithm, with a gain of 0.3252.

Data analysis reveals strong correlations between certain parameters (e.g., depth and geological characteristics) and rockburst grade in shallow engineering regions. Addressing these correlations poses a crucial challenge, and future research endeavors should focus on leveraging multi-source, multi-phase data to deepen this study understanding of rockburst occurrences and the underlying causal factors. This deeper understanding will inform the refinement and enhancement of explosion prevention and pre-blast programs, ultimately improving safety measures in shallow underground engineering endeavors.

## Data Availability

All data that support the findings of this study are available from the corresponding author upon reasonable request.
